# Evaluating Effects of Wrinkle Defects on Impact Response and Residual Compressive Strength After Impact in CFRP

**DOI:** 10.3390/polym17101355

**Published:** 2025-05-15

**Authors:** Jian Wang, Huiming Ding, Shidi Zhang, Han Wang, Yunbo Bi, Zhengli Hua

**Affiliations:** 1 Donghai Laboratory, Zhoushan 316021, China; 2 Institute of Advanced Equipment, College of Energy Engineering, Zhejiang University, Hangzhou 310027, China; 3 College of Mechanical Engineering, Zhejiang University, Hangzhou 310027, China; 4 Hydrogen Energy Institute, Zhejiang University, Hangzhou 310027, China

**Keywords:** CFRP, wrinkle defect, impact response, compressive strength after impact

## Abstract

Carbon fiber-reinforced polymer (CFRP) has become widely applied in engineering fields such as aerospace and the automotive industries. Evaluating the damage tolerance of CFRP with manufacturing defects under impact loads is crucial in ensuring the reliable service of CFRP components. In this study, four types of wrinkle defects are designed, and the effect mechanism is thoroughly discussed, focusing on the impact and compressive response. The results indicate that the wrinkle defects primarily affect the impact response via the wrinkle fibers being subjected to impact stress and wrinkle stress concentration. Notably, the first peak contact force of the specimen with a wrinkle at the 12th layer is reduced by approximately 20.00% compared to that of the specimen with a wrinkle at the third layer. Additionally, the first peak contact force of the specimen subjected to a reverse impact direction decreases by about 14.00% compared to that under a forward impact direction. The impact direction also plays a significant role in the impact response by altering the loading conditions of the wrinkle fibers during impact. Regarding the compressive performance after impact, specimens with a wrinkling layer close to the impact surface show a slight 4.80% increase in residual compressive strength, which is attributed to the greater suppression of impact damage by the wrinkle fibers. However, all other specimens with wrinkle defects demonstrate varying degrees of reduction in residual compressive strength after impact compared to the specimens without wrinkle defects. The maximum reduction is approximately 27.50% for specimens subjected to a reverse impact direction. Furthermore, the amplitude of the decrease in the residual compressive strength is mainly determined by the matrix damage and delamination that occur during impact.

## 1. Introduction

Polymer composites have attracted substantial interest in recent years due to their remarkable strength-to-weight ratio and stiffness. Driven by performance requirements and environmental durability considerations, composites are increasingly using natural fibers, synthetic fibers, and hybrid combinations as reinforcements. As one of the most emphasized lightweight and high-strength composites, carbon fiber-reinforced polymer (CFRP) is widely applied in engineering fields such as aerospace and the automotive industries [1,2,3,4,5,6,7]. However, it is important to note that defects like porosity, bridging, and wrinkles are common in the manufacturing process of CFRP. These defects can negatively impact the reliability of composite structural components [8,9,10]. Additionally, CFRP is highly sensitive to impact loads, and manufacturing defects in CFRP can lead to catastrophic structural failure, even under small impact loads [11,12,13]. Therefore, evaluating the damage tolerance of CFRP with defects under impact loads is essential to ensure the reliable service of CFRP structural components [14,15].

A wrinkle is the most common and critical manufacturing defect in composites. It arises from a complex interplay of mechanical, thermal, and material factors. The primary reasons can be categorized into process-induced factors (such as uneven pressure distribution during layup/consolidation, improper tool–part interaction causing shear stress, excessive autoclave/vacuum bag pressure leading to fiber buckling, etc.), material characteristics (such as low ply tack strength promoting inter-ply slippage, high resin viscosity impeding proper fiber alignment, etc.), design-related issues (such as sharp curvature regions (R < 5 mm) inducing compressive stress, ply drop-off configurations creating thickness transitions, etc.), and thermal effects (such as mismatched thermal expansion coefficients between fibers and the matrix, rapid heating rates (>3 °C/min) causing resin flow instability, etc.) [16,17,18]. Some researchers have conducted studies to assess the effects of wrinkle defects on the degradation of the mechanical performance of the composite. As for the tensile properties, studies indicate that shear stress is typically concentrated at wrinkles, which leads to noticeable matrix cracks and delamination before tensile failure, resulting in a maximum reduction in tensile strength of approximately 40%. Moreover, the effect of wrinkles on the damage mechanism is also related to the wrinkle angle [19,20]. Compared to the tensile properties, wrinkle defects have a more significant effect on the compressive properties of the composite. This is mainly because the tensile strength of the composite is usually determined by the fibers, whereas the compressive strength is primarily influenced by the matrix strength and the occurrence of delamination. During compressive loading, wrinkles mainly cause matrix cracks and delamination, leading to noticeable degradation in compressive strength, amounting to 73% [21,22,23]. Additionally, relevant studies show that the compressive strength of CFRP specimens decreases with an increase in the wrinkle angle. Specimens with smaller wrinkle angles tend to fail via the torsional mode, whereas those with larger wrinkle angles exhibit the through-thickness splitting failure mode [24,25]. Some researchers have also focused on the effects of wrinkle defects on the fatigue performance of the composite. Under tensile–tensile cyclic loading, the wrinkle defects in specimens have an adverse effect on the occurrence of early delamination. At a given load, the fatigue life of specimens with wrinkle defects is approximately an order of magnitude lower than that of specimens without wrinkle defects [26]. Under compressive–compressive cyclic loading, the fatigue damage mechanism of specimens with wrinkle defects is dominated by the propagation of small shear cracks in the matrix. Wrinkles significantly influence the damage evolution and fatigue life of specimens. The fatigue life of a specimen with out-of-plane waviness is reduced to approximately 50% of the life of the no-defect material. Moreover, the damage modes, damage evolution, and fatigue life of specimens with wrinkle defects differ between tensile–compressive and compressive–compressive cyclic loading conditions [27,28,29].

In summary, although researchers have conducted some studies on the effects of wrinkle defects in the degradation of the mechanical performance of composites, very few studies have focused on the effects of wrinkle defects under impact loads. As a result, the quantitative influence mechanisms of wrinkle defects on the impact performance of composites remain unclear, which hinders reliable structural design. Therefore, the primary objective of this study is to quantitatively evaluate the impact response and residual compressive strength after impact in CFRP specimens with different types of wrinkle defects. Correspondingly, compared with current studies focusing on the effects of wrinkle defects on the mechanical properties, the novelty of this study lies in the quantification and systematic analysis of different types of wrinkle defects on the impact performance. The evaluation is conducted through both experiments and numerical simulations. The study examines four key variables related to wrinkle defects: the wrinkle offset distance, wrinkling layer, wrinkle number, and impact direction. The effects of these wrinkle defects are thoroughly discussed based on the experimental and numerical simulation results, focusing on the impact response, compressive response after impact, and associated damage mechanisms. The findings of this study can provide valuable insights in assessing impact damage tolerance in composite materials and contribute to a better understanding of the effects of manufacturing defects.

## 2. Materials and Methods

### 2.1. Test Matrix and Wrinkle Defect Characterization

**Figure 1:**
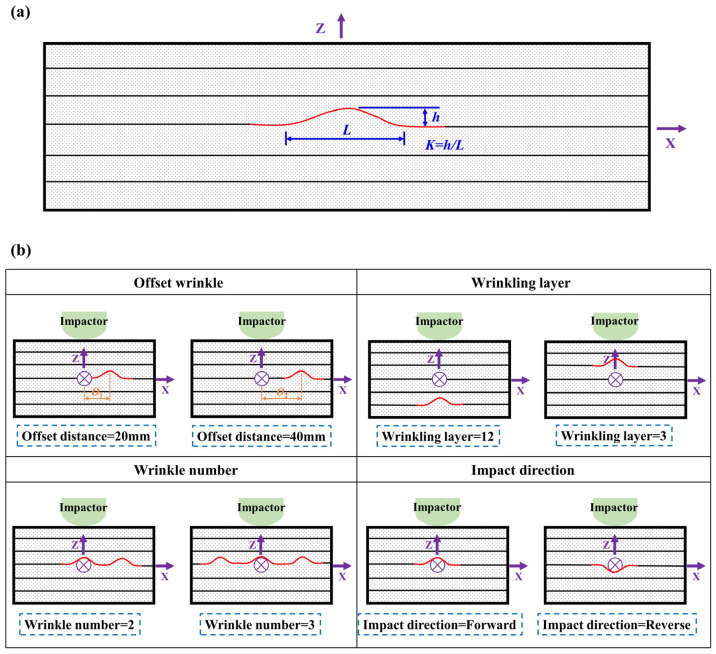
Schematic of (**a**) wrinkle ratio definition; (**b**) wrinkle defect characterization.

The wrinkle ratio *k* in this study is defined as the ratio of the wrinkle amplitude *h* to the wrinkle width *L*, as illustrated in Figure 1a and Equation (1). Four key variables related to wrinkle defects are evaluated in this study: the wrinkle offset distance, wrinkling layer, wrinkle number, and impact direction. The test matrix is presented in Table 1. The schematic of wrinkle defect characterization for the specimen is depicted in Figure 1b. The wrinkle offset distance refers to the distance between the wrinkle centerline and the center of the specimen. The wrinkling layer is the layer where wrinkles start from the impact surface. To examine the effects of different wrinkling layers, the middle or 8th layer is selected a reference for comparison. Additionally, to ensure the manufacturability of specimens with wrinkle defects, the 3rd layer, near the forward impact position, and the 12th layer, further away from the forward impact position, are also selected as wrinkling layers. As for the wrinkle numbers, the specimen with one wrinkle is adopted as the reference because it represents the most straightforward influence mechanism. Furthermore, to discuss the effect of the wrinkle number, specimens with two and three wrinkles are also designed. The inclusion of two wrinkles could introduce a symmetrical condition, while three wrinkles may lead to a more complex influence mechanism. The forward impact direction is defined as the direction of the impact surface adjacent to the protruding side of the wrinkle, as illustrated in Figure 1b. In the authors’ previous study, the effects of the wrinkle ratio and impact energy on both the impact response and the compressive response after impact have been analyzed. The results indicate that a large wrinkle has a more significant effect on the impact response and the compressive response after impact in CFRP specimens compared to small and medium wrinkles under impact energy of 15 J. Therefore, this study specifically focuses on the effects of various wrinkle defects by selecting a large wrinkle with a wrinkle ratio of 0.075 and impact energy of 15 J to ensure a noticeable difference in the impact responses and compressive responses of specimens with different wrinkle defects.

(1) $$k=\frac{h}{L}$$

### 2.2. Experimental Setup

**Figure 2:**
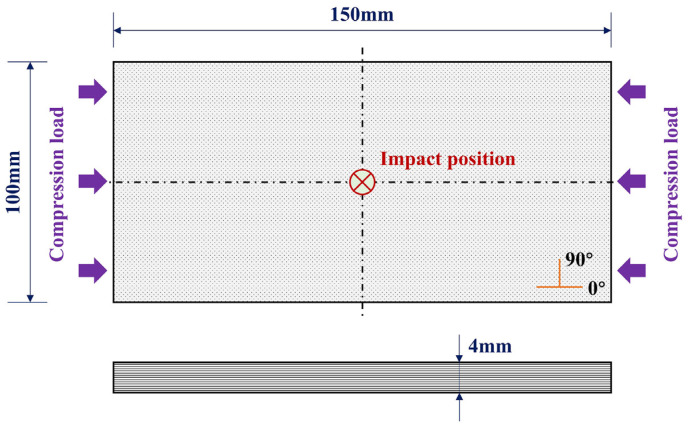
Schematic of specimen geometry.

The CFRP used in this study is T700/7901 with a single-layer thickness of 0.25 mm. The material properties are listed in Table 2. To conduct the compressive tests after impact (CAI), the specimens are designed as flat plates with dimensions of 150 mm × 100 mm × 4 mm, in accordance with ASTM D7136 [30], as shown in Figure 2. The lay-up sequence is [0°/90°]_4s_.

**Figure 3:**
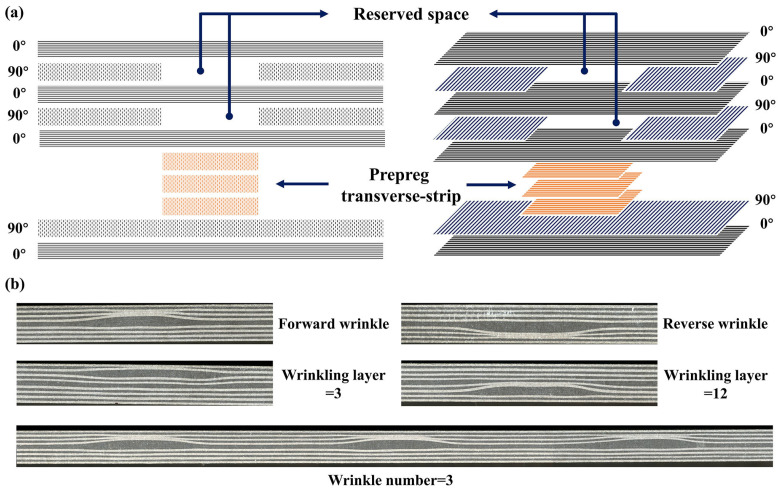
(**a**) Schematic of prefabrication of wrinkle defect; (**b**) micro-morphologies of specimens with wrinkle defects.

In terms of specimen preparation, the precision of the wrinkle defect is crucial for this study. The transverse-strip method is commonly used to prefabricate wrinkle defects by inserting the prepreg transverse strip between different prepreg layers. However, specimens prepared by this method often exhibit noticeable bulges on their surfaces. In the manufacturing process, products with bulges are classified as unqualified, rendering any study on such specimens less meaningful due to their significant deviation from reality. Moreover, some typical composite structures (such as the cylindrical shell) can develop wrinkle defects even if the surface is flat. Therefore, it is more realistic to study wrinkle defects in specimens with a flat surface. To address this issue, this study adopts a wrinkle defect pre-fabrication method modified from the “strip method” and “void method”. This method can guarantee that the specimens have a smooth surface. The basic principle of this method involves inserting prepreg transverse strips, made from the same material as the specimen, and leaving gaps during the composite layering process, as illustrated in Figure 3a. Moreover, different wrinkle ratios can be achieved by controlling the number of inserted prepreg transverse strips, and the surface flatness of the specimen with wrinkle defects is controlled by adjusting the number of blanks. During specimen preparation, the fiber height is increased by embedding the prepreg transverse strips, and effective volume compensation is achieved by cutting voids in the adjacent 90° lay-up. This modification can avoid the surface bulging issue caused by embedding prepreg transverse strips when using the conventional “strip method” for wrinkle fabrication. Based on previous experiments, the wrinkle ratio of 0.075 can be achieved by embedding three transverse prepreg strips with a thickness of 0.25 mm along with two voids in the 90° lay-up, as shown in Figure 3a. The micro-morphologies of the specimens with wrinkle defects prepared with the above method are shown in Figure 3b. The average error between the actual and designed wrinkle ratios is 10.67%, indicating that the fabricated wrinkle defects exhibit good precision.

**Figure 4:**
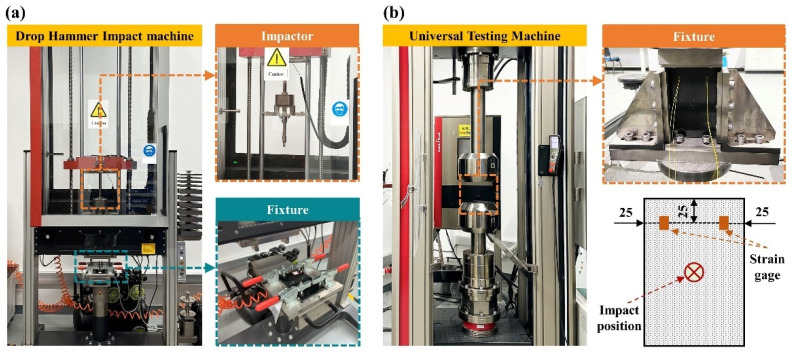
Mechanical test of (**a**) impact; (**b**) CAI.

For the impact test, in accordance with ASTM D7136 [30], the flat specimen shown in Figure 2 is fixed on a drop-weight impact testing machine using a special fixture, as illustrated in Figure 4a. The specimen is fixed to the support by the fixture, and a 12.7 mm diameter hemispherical impactor is used. During the test, the impact energy is controlled by adjusting the height from which the impactor freely falls along the guide rail. As for the compressive test after impact, in accordance with ASTM D7137M [32], a universal testing machine is adopted, as shown in Figure 4b. To ensure that the specimen is subjected to a uniform compressive load during the test, a pre-load is applied to the specimen before the compressive test. In this study, the pre-load is set at 10% of the expected failure load. Subsequently, the specimen is gradually unloaded to approximately 150 N to complete the pre-loading process. The compressive loading rate is set at 1.25 mm/min. During pre-loading, the strain gauge shown in Figure 4b is used to monitor the strain response. The maximum curvature percentage during pre-loading is calculated using Equation (1). If the curvature percentage exceeds 10%, adjustments are made to the specimen’s position in the fixture, and the pre-loading procedure is repeated to avoid localized uneven loading during the subsequent compressive tests.

(2) $$C=\frac{\varepsilon_{1}-\varepsilon_{2}}{\varepsilon_{1}+\varepsilon_{2}}\times 100\%$$

In Equation (2), *C* represents the curvature percentage of the specimen, and *ε*_1_, *ε*_2_ are the strains on the front and back surfaces of the specimen, respectively. The compressive test is completed when the load decreases to below 70% of the peak load. The residual compressive strength after impact is calculated using Equation (3).

(3) $$\sigma_{CAI}=\frac{F}{h_{s}\times w}$$

In Equation (3), *σ_CAI_* represents the residual compressive strength after impact, *F* is the peak load, and *h_s_* and *w* are the thickness and width of the specimen, respectively.

## 3. Effect of Wrinkle Defects on Impact Response

### 3.1. Numerical Model

To accurately model wrinkle defects in the numerical simulation, the cosine trigonometric method proposed by Daniel and Hsiao [33] is adopted in this study to characterize the wrinkle defects, as shown in Equation (4).

(4) $$y=h\cos(\frac{\pi x}{L})+b$$

In Equation (4), *x* and *y* represent the coordinates of the wrinkle in the X- and Y-axis directions, respectively; *h* is the wrinkle amplitude; *L* is the wrinkle width; and *b* is the coordinate of the current layer in the Z-axis direction. The parametric modeling of the wrinkle defect is implemented based on the Python language, version of 3.12.

**Figure 5:**
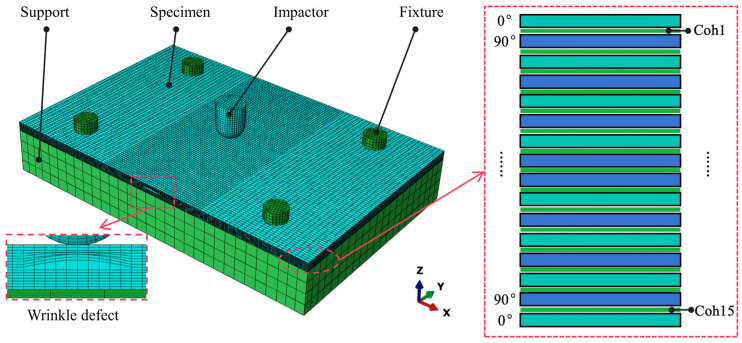
Numerical model of impact of CFRP specimen with wrinkle defect.

Based on the parametric modeling method for the wrinkle defect, the numerical simulation model for the impact test of the CFRP specimens with wrinkle defects is developed using the ABAQUS 2022 software, as shown in Figure 5. This model includes the CFRP specimen with a wrinkle defect, the impactor, the fixture, and the support. An essential aspect of impact simulation is the selection of failure criteria for composite materials, as it significantly influences the accuracy of the results.

The PUCK criteria enhance the damage assessment of the matrix by incorporating factors such as the fracture angle and effect of friction, making it more suitable for failure prediction with significant matrix damage, such as in impact simulation [34,35]. Consequently, in this study, the PUCK failure criteria are employed to effectively simulate fiber and matrix damage within the CFRP specimen, balancing accuracy and efficiency. Relevant material properties are summarized in Table 2. Additionally, zero-thickness cohesive elements are inserted between adjacent layers of the CFRP specimen to simulate delamination during the impact process. Based on the relevant experimental research on the interfacial properties of the same CFRP material in this study [36,37,38,39], all cohesive parameters regarding the interfacial strength and fracture toughness are determined, as shown in Table 3. In addition, as for the interfacial stiffness *K* shown in Table 3, it is generally experience-dependent and differs among researchers. However, the interfacial stiffness should be sufficiently large to minimize the elastic energy stored, while being sufficiently small to avoid numerical ill-conditioning. In this study, the interfacial stiffness value is estimated as 50,000 MPa/mm using the equation proposed by Turon et al. [40], as shown in Equation (5):

(5) $$K_{i}=\frac{\alpha E_{33}}{t}(i=I,\mathrm{II},\mathrm{III})$$

where *E*_33_ is the Young’s modulus of the laminates in the thickness direction, and *t* is taken as the total thickness of the laminates in this study, i.e., α = 25, as recommended. The CFRP specimen, impactor, and fixture are all modeled using C3D8R elements, while the cohesive elements are modeled with COH3D8R. To prevent the hourglass effect that can occur with C3D8R elements and to enhance the accuracy of the simulation, a mesh sensitivity analysis is conducted. The mesh size around the impact region (wrinkle area) is set to 1 mm to refine the mesh, while the global mesh size is set to 2 mm. The CFRP specimen is assembled between the support and the fixture. General contact is employed to simulate the interactions among the impactor, specimen, support, and fixture. The contact friction coefficient is set to 0.3, and fixed boundary conditions are applied to both the support and the fixture to simulate the contact relationships during the impact process. To simulate the impact from the impactor, the mass of 6.615 kg is assigned to it, and its degrees of freedom in the direction perpendicular to the specimen surface are released. The vertical impact of the impactor is simulated using the predefined velocity field. To verify the accuracy of the numerical simulation model, the impact simulation results of all the specimens are compared with the experimental results, as described in the Appendix A.

### 3.2. Impact Response

**Figure 6:**
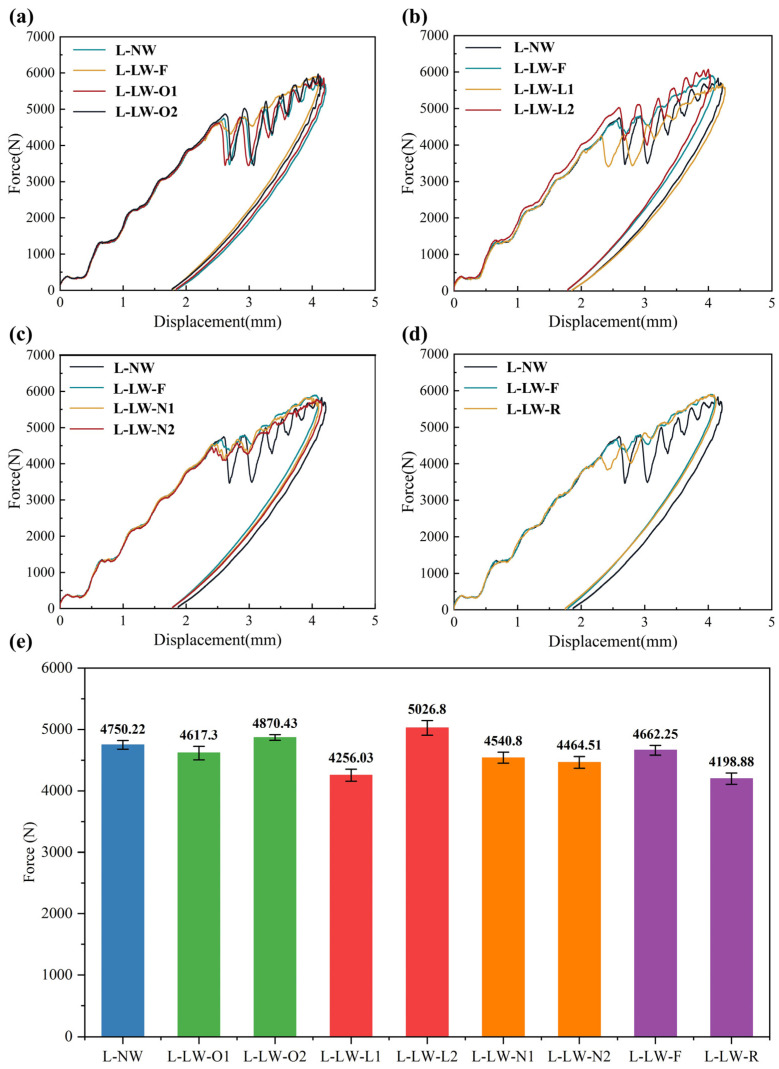
Contact force–displacement curves of CFRP with different wrinkle defects: (**a**) offset distance; (**b**) wrinkling layer; (**c**) wrinkle number; (**d**) impact direction; (**e**) first peak contact force.

The contact force–displacement curves for the impact responses of various specimens with different wrinkle defects are presented in Figure 6. To prevent overlapping curves, the contact force–displacement curves for each type of specimen are plotted separately. These curves represent the average results of five specimens. Figure 6a displays the impact response curves for the specimens with wrinkle offset distances of 20 mm and 40 mm. Notably, the impact response curves for the specimens with offset wrinkles are similar in form to the typical impact response curves of specimens without wrinkles. The curves exhibit a sharp decrease after reaching the first peak contact force, followed by significant oscillations. In contrast, the impact response curve for the specimen with the wrinkle situated at the center exhibits a relatively small decrease after the first peak contact force, as well as smaller oscillations in the subsequent stages. The primary reason for this phenomenon can be attributed to the impact stress experienced by the wrinkle fibers. In specimens with centrally located wrinkles, the wrinkle defect is positioned at the center of the impact location, and the protruding part of the wrinkle is close to the impact surface. As a result, the wrinkle fibers can be partially subjected to stress caused by the impact load, which helps to suppress damage to the matrix. Consequently, this leads to a significant reduction in both the sharp decrease and the subsequent oscillation amplitude of the impact response curve. In contrast, for specimens with wrinkles positioned 20 mm and 40 mm away from the center, the stress induced by the impact load on the wrinkle fibers is relatively small. As a result, their effectiveness in suppressing matrix damage is reduced, leading to a larger sharp decrease and greater subsequent oscillations in the impact response curve. This behavior is similar to that of specimens without wrinkles.

The impact response curves for specimens with different wrinkling layers are displayed in Figure 6b. As previously mentioned, the impact response of the specimen with a center wrinkle is affected by the mechanism by which wrinkle fibers are subjected to the stress induced by the impact load, resulting in a slight decrease and oscillations in the impact response. In contrast, for the specimens with wrinkles located at the 3rd and 12th layers, although the dimensions and locations of the wrinkles are identical to those of the specimens with center wrinkles, the impact response curves still exhibit a significant sharp decrease and greater oscillations after the first peak contact force. Additionally, the peak contact forces for specimens with different wrinkling layers also show notable differences. Specifically, the first peak contact force for the specimen with a wrinkle at the 12th layer is reduced by approximately 20% compared to the specimen with a wrinkle at the third layer. The differences in the wrinkling layers may be related to the dominant stress in the regions where the wrinkles are located. During the impact process, the stress in the specimen typically exhibits “upward compression, downward tension” behavior. When the wrinkle is situated at the third layer, which is close to the impact surface, the wrinkle fibers are subjected to a significant portion of the stress induced by the impact load. As a result, the peak contact force is not significantly lower than that of the central wrinkle specimen and may even slightly increase. However, since the wrinkle is located in the region dominated by compressive stress, the matrix crack propagates quickly under compressive stress, leading to a significant decrease and oscillations in the impact response curve. Conversely, when the wrinkle is located at the 12th layer, far away from the impact surface, the wrinkle fibers are subjected a smaller portion of the stress induced by the impact load. Additionally, the matrix in the wrinkle region is subjected to tensile stress, which causes premature matrix damage. This leads to a noticeable reduction in the peak contact force and greater oscillations in the response curve.

The impact response curves of specimens with different numbers of wrinkle defects are shown in Figure 6c. It can be found that the number of wrinkles has little effect on the overall shape of the impact response curve. All specimens with wrinkle defects show a slight decrease and oscillations after reaching the first peak contact force, regardless of the number of wrinkles. This behavior can be attributed to the fact that all specimens contain wrinkles located at the center of the impact location, regardless of the number of wrinkles. As mentioned above, the impact stress experienced by the wrinkle fibers leads to a small decrease and oscillations in the impact response curve. However, the number of wrinkles has a certain influence on the first peak contact force of the impact response curve. As the number of wrinkles increases, the peak contact force tends to decrease slightly. This may be because the increase in the number of wrinkles induces more stress concentration areas, leading to premature matrix damage and the delamination of the specimen under the impact load.

The impact response curves of specimens with wrinkle defects under different impact directions are shown in Figure 6d. It can be found that the impact direction also has a noticeable effect on the impact responses of specimens with wrinkle defects. Specifically, compared to the specimen without wrinkles, although the response curve of the specimen under a reverse impact direction also shows a small decrease and oscillations after reaching the first peak contact force, the amplitude of oscillation is noticeably more severe than that of the specimen under a forward impact direction. Furthermore, the first peak contact force of the specimen under a reverse impact direction is approximately 14% lower than that of the specimen under a forward impact direction. In the case of the specimen under a reverse impact direction, the impact surface is located on the concave side of the wrinkle. This means that the wrinkle fibers cannot effectively mitigate the stress induced by the impact load. Consequently, the stress concentration caused by the wrinkle leads to premature damage during the impact process, resulting in a significant reduction in the first peak contact force.

### 3.3. Damage Analysis

**Figure 7:**
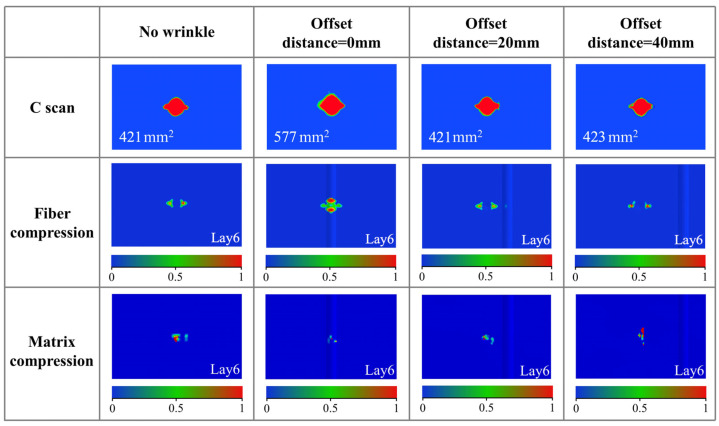
Damage analysis of CFRP plate with different offset distances.

To further discuss the damage of specimens after impact loading, the stress and damage of the specimens are analyzed through C-scan and progressive damage numerical simulations. The damage of specimens with different wrinkle offset distances is shown in Figure 7. It can be found that the delamination area of specimens with wrinkles at the impact location is the largest, with an approximately 37% increase in the delamination area compared to that of the specimen without wrinkle defects. The significant increase in the delamination area can be attributed to the impact stress experienced by the wrinkle fibers. The wrinkle fibers are subjected to the stress induced by the impact load, leading to relatively high shear stress between the wrinkle layers. With the influence of shear stress, delamination is more likely to occur. Interestingly, the delamination area of specimens with offset wrinkles shows no significant difference compared to that of the specimens without wrinkle defects, and it is not affected by the offset distance. This is similar to the previously mentioned impact response curves. When the wrinkle is offset from the impact point, the wrinkle fibers are unable to effectively mitigate the stress induced by the impact load. This results in the delamination area being similar to that of the specimens without wrinkle defects. Meanwhile, the compressive damage and the stress distribution further confirm the influence mechanism of the wrinkle offset distance on the impact responses of the specimens. Conversely, when the wrinkle is located at the impact position, the wrinkle fibers are subjected to the stress induced by the impact load, leading to relatively severe fiber compression damage and stress concentration at the impact point. Additionally, the impact stress experienced by the wrinkle fibers could suppress matrix damage, resulting in less matrix compressive damage. In contrast, as for the specimens without wrinkle defects and with offset wrinkles, since no effective wrinkle fibers are subjected to the stress induced by the impact load at the impact point, the fiber compression damage at the impact point is relatively small, while the matrix compressive damage is more severe under the impact load. Therefore, the effect of the wrinkle offset distance on the impact response is primarily determined by the role of the wrinkle fibers in mitigating the stress induced by the impact load.

**Figure 8:**
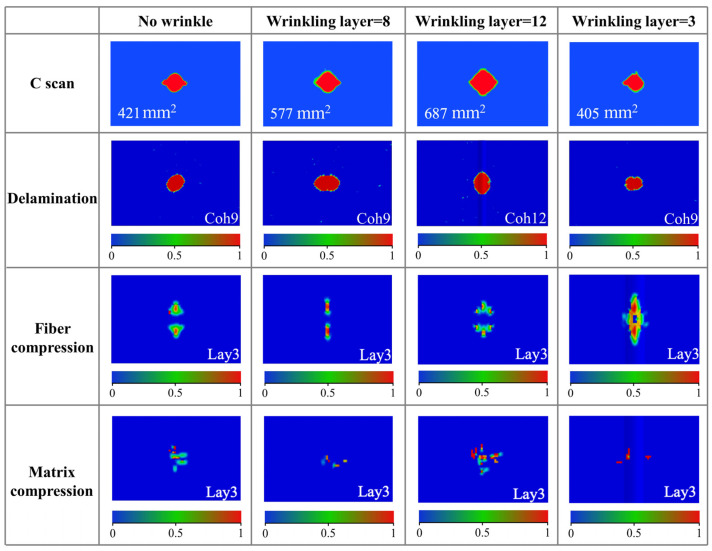
Damage analysis of CFRP plate with different wrinkling layers.

The damage of specimens with different wrinkling layers is shown in Figure 8. As for the delamination areas obtained through C-scan, the delamination area of the specimen shows a noticeable increasing trend as the wrinkling layer moves farther from the impact surface. Specifically, the specimen with the wrinkle at the 12th layer exhibits the largest delamination area, which is approximately 63% larger than that of the specimen without wrinkle defects. The specimen with the wrinkle at the eighth layer shows a 37% increase in the delamination area, while the specimen with the wrinkle at the third layer shows a 4% decrease in the delamination area. Moreover, the simulation results indicate that the location of the maximum delamination area varies with the position of the wrinkling layer. The position of maximum delamination of the specimens with wrinkles at the eighth layer and the third layer is near the neutral plane (Coh 9), while that of the specimen with a wrinkle at the 12th layer occurs at the wrinkling layer (Coh 12). The phenomenon described above is also determined by the role of the wrinkle fibers in mitigating the stress induced by the impact load and the stress concentration caused by the wrinkle fibers. As for specimens with wrinkling layers far from the impact surface, the fiber compressive damage is relatively small, while the matrix compressive damage is larger. This indicates that the wrinkle fibers are subjected to less of the impact load, and the stress concentration caused by the wrinkle fibers plays a dominant role, resulting in a larger delamination area under an impact load. In contrast, as for specimens with wrinkling layers close the impact surface, the fiber compressive damage is more pronounced, while the matrix compressive damage is smaller. This indicates that the wrinkle fibers play a dominant role in mitigating the stress caused by the impact load and suppressing matrix damage, leading to a smaller delamination area. Therefore, the effect of the wrinkling layer on the impact response depends on the dominant role between the load-bearing capacity of wrinkle fibers under an impact load and the stress concentration induced by the wrinkle fibers.

**Figure 9:**
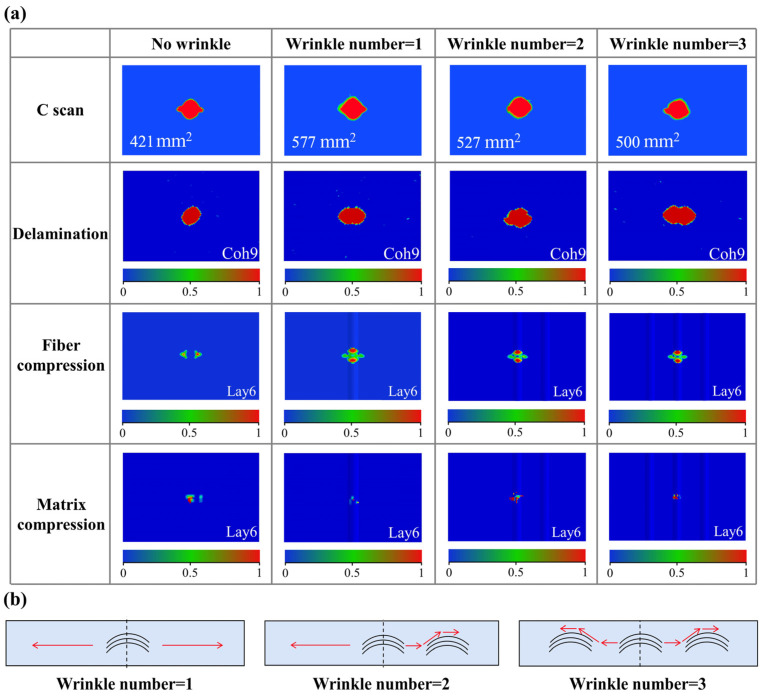
(**a**) Damage of CFRP plate with different wrinkle numbers; (**b**) schematic of delamination of CFRP plate with different wrinkle numbers.

The damage of specimens with different wrinkle numbers is shown in Figure 9a. The C-scan results reveal that the delamination area of specimens with wrinkle defects is significantly larger than that of specimens without wrinkle defects. As mentioned above, the increase in the delamination area of specimens with wrinkle defects is attributed to the shear stress between the wrinkle layers and localized stress concentration. However, compared to the specimen with a single wrinkle, the delamination areas of specimens with two and three wrinkles decrease by approximately 9% and 13%, respectively. Moreover, the simulation results show that the maximum delamination area is located in the middle wrinkling layer, which is not affected by the number of wrinkles. This phenomenon may be determined by the energy dissipation relationship between matrix damage and delamination propagation, where part of the impact energy introduced by the impact load is dissipated through matrix damage and delamination propagation. As shown in Figure 9b, as for the specimen with a single wrinkle defect, the delamination propagation mainly follows the straight fiber layer path. When matrix damage occurs at the wrinkle fibers, the specimen is more susceptible to delamination damage. Consequently, most of the impact energy is dissipated by delamination, resulting in smaller matrix compressive damage. As for the specimen with two wrinkle defects, shown in Figure 9b, delamination propagates from the center impact point to both sides. On the side without wrinkle fibers, the delamination expansion dissipates less energy, making the specimen more susceptible to further delamination. Conversely, on the side with wrinkle fibers, the increased resistance to delamination propagation leads to greater energy dissipation through the delamination process. Consequently, delamination is less likely to occur on the side with wrinkle fibers compared to the side with straight fibers. Instead, energy is dissipated through matrix compressive damage, as shown in Figure 9a. Similarly, as for specimens with three wrinkle defects, since wrinkle fibers are located on both sides of the center impact point, the resistance to delamination propagation is greater than that of specimens with two wrinkle defects, resulting in the smallest delamination area and more noticeable matrix compressive damage. Therefore, under the condition that wrinkle fibers are located at the impact point, the wrinkle number primarily affects the impact damage through the matrix damage at the impact point and the energy dissipation relationship between delamination propagation and matrix damage at non-center impact points.

**Figure 10:**
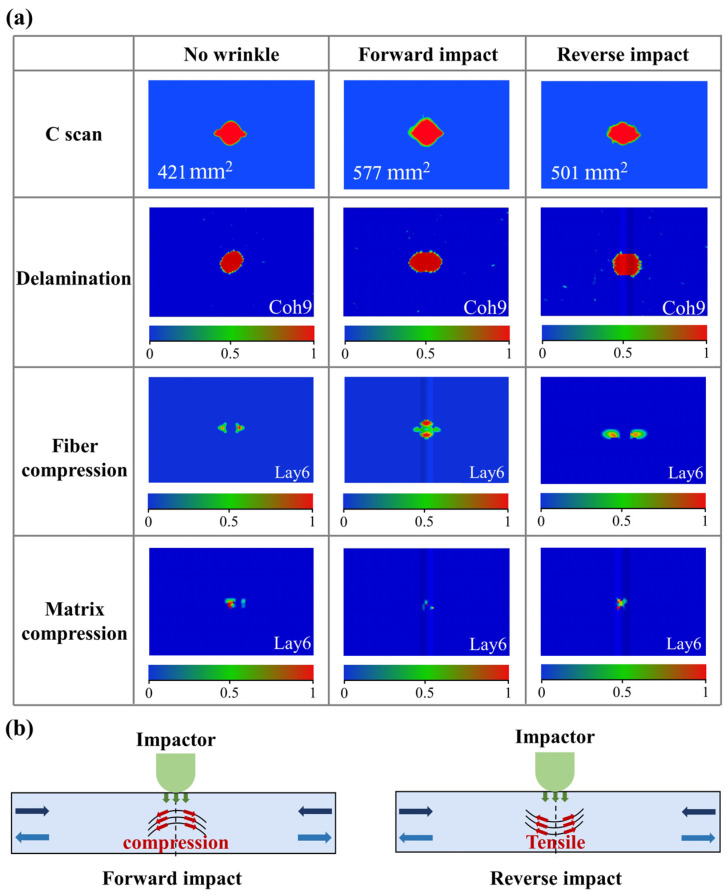
(**a**) Damage of CFRP plate with different impact directions; (**b**) schematic of load analysis of CFRP plate with different impact directions.

The damage of specimens under different impact directions is shown in Figure 10a. Both the C-scan and numerical simulation results indicate that the delamination areas of specimens with wrinkle defects are larger than those of specimens without wrinkle defects. Moreover, the delamination areas of specimens under the reverse impact direction are approximately 13% smaller than those of specimens under the forward impact direction. This difference in the delamination area is primarily determined by the different loading conditions of the wrinkle fibers during the impact process. As shown in Figure 10b, when the specimen undergoes forward impact, the convex side of the wrinkle fibers is close to the impact surface, which results in these wrinkle fibers mainly being subject to compressive loads during the impact process. Consequently, the interlaminar shear stress induced by the load bearing of the wrinkle fibers, combined with local stress concentration in the wrinkle fibers, leads to a higher tendency for large delamination. In contrast, when the specimen undergoes a reverse impact, the concave side of the wrinkle fibers is near the impact surface. As shown in Figure 10b, during the impact process, the wrinkle fibers mainly bear a tensile load, and the wrinkle fibers are no longer significantly subjected to the stress induced by the impact load. As a result, the specimen experiences smaller fiber compression damage and larger matrix compression damage. Thus, the delamination is mainly caused by the local stress concentration in the wrinkle fibers. Overall, the influence mechanism of the impact direction on the impact response is determined by the different loading conditions of the wrinkle fibers during the impact process.

## 4. Effects of Wrinkle Defects on CAI

### 4.1. Compressive Response

**Figure 11:**
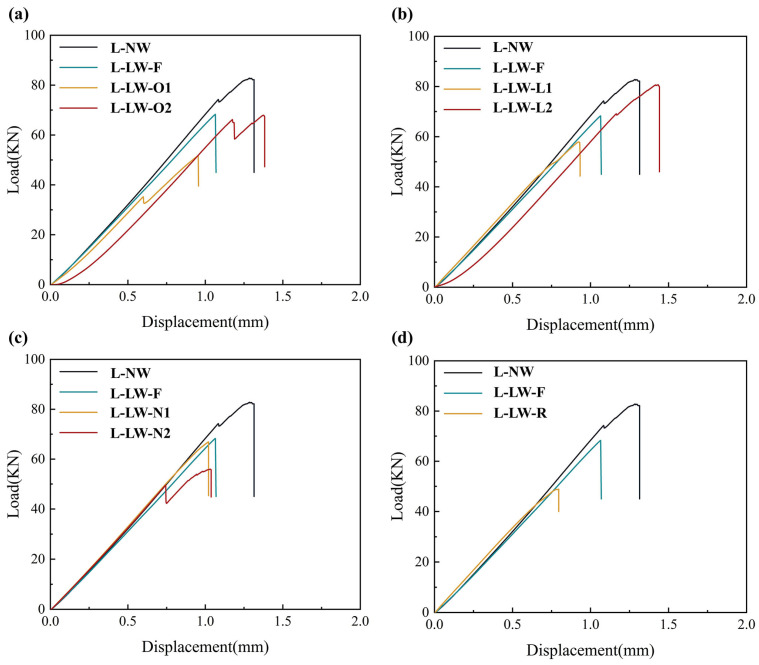
Compressive load–displacement curves of specimens with different (**a**) offset distances; (**b**) wrinkling layers; (**c**) wrinkle numbers; and (**d**) impact directions.

The load–displacement curves and residual compressive strength for each type of specimen after impact compressive tests are shown in Figure 11 and Table 4, respectively. The residual compressive strength is calculated using Equation (3), and the “variation” in Table 4 represents the relative change in the residual compressive strength of the specimens with different wrinkle defects compared to that of the specimen without wrinkle defects.

The wrinkle offset distance exhibits a significant effect on the residual compressive strength after impact, as shown in Table 4. Compared to the specimen without wrinkle defects, the residual compressive strength after impact in specimens with wrinkle offset distances of 0 mm, 20 mm, and 40 mm decreases by approximately 2.90%, 15.10%, and 7.90%, respectively. This difference is primarily determined by the matrix damage after impact. As discussed in Section 3.3, when the wrinkle fibers are positioned at the impact point, the fact that they are subjected to the impact load results in smaller matrix damage after impact. Consequently, the specimen can withstand relatively larger compressive loads during the loading process, leading to a smaller reduction in its residual compressive strength. Conversely, in terms of specimens with offset wrinkles, the reduced extent to which the wrinkle fibers are subjected to the impact load results in more noticeable matrix damage after impact, leading to a significant reduction in the residual compressive strength. Additionally, compared to the specimen with a 20 mm offset wrinkle, the specimen with a 40 mm offset wrinkle shows an approximately 8.50% increase in residual compressive strength after impact. This may be because, when the wrinkle is closer to the impact point, the local stress concentration introduced by the wrinkle makes delamination more likely to propagate from the center impact point to the sides of the specimen during compression, resulting in lower residual compressive strength.

As for specimens with different wrinkle layers, compared to the specimen without wrinkle defects, the residual compressive strength of specimens with wrinkles at the 8th and 12th layers decreases by approximately 2.90% and 26.00%, respectively. Interestingly, the residual compressive strength of the specimen with the wrinkle at the third layer increases by about 4.80%. When the wrinkle is at the 12th layer, the wrinkle is farther from the impact surface, resulting in the wrinkle fibers being less subjected to the impact load. This leads to more significant matrix damage and delamination. Consequently, the residual compressive strength after impact exhibits a considerable decrease. In contrast, when the wrinkle layer is closer to the impact surface, such as in the specimen with the wrinkle at the eighth layer, the extent to which the wrinkle fibers are subjected to the impact load increases. This results in relatively less matrix damage and delamination. As a result, the residual compressive strength shows an increasing trend compared to specimens with wrinkle layers farther from the impact surface. Furthermore, when the wrinkle layer is even closer to the impact surface, such as in the specimen with the wrinkle at the third layer, the wrinkle fibers are more subjected to the impact load, which leads to smaller matrix damage and delamination after impact. As shown in Figure 8, the matrix damage and delamination of the specimen with the wrinkle at the third layer are even smaller than in the specimen without wrinkle defects. Consequently, the residual compressive strength after impact in the specimen with the wrinkle at the third layer increases by approximately 4.80% compared to that of the specimen without wrinkle defects.

As for specimens with different wrinkle numbers, compared to the specimen without wrinkle defects, the residual compressive strength after impact decreases significantly with an increase in the wrinkle number. Specifically, the residual compressive strength after impact in specimens with one, two, and three wrinkles decreases by approximately 2.90%, 4.20%, and 26.40%, respectively. This is related to the increased sensitivity to delamination caused by the local stress concentration introduced by the wrinkle defect under compressive loading. As the wrinkle number increases, the matrix damage after impact also shows a slight increase, making the specimens more vulnerable to failure during the subsequent compression process. Furthermore, with the increase in the wrinkle number, the local stress concentration caused by the wrinkle defects makes the specimen more prone to delamination under compressive loading. Consequently, the residual compressive strength after impact in the specimen with three wrinkles is reduced by approximately 26.40% compared to that of the specimen without wrinkle defects.

As for specimens with different impact directions, the impact direction has a significant effect on the residual compressive strength after impact in specimens with wrinkle defects, as shown in Figure 11 and Table 4. Compared to the specimen without wrinkle defects, the residual compressive strength after impact in the specimen with a wrinkle defect under a forward impact decreases by approximately 2.90%, while that of the specimen with a wrinkle defect under a reverse impact decreases by up to 27.50%. This is mainly determined by the damage to the fibers and matrix after impact. As shown in Figure 10, in terms of the specimens with wrinkles under a reverse impact, the wrinkle fibers mainly bear tensile loading during the impact, and the relatively smaller extent to which they are subjected to the impact load leads to more severe matrix damage and delamination. Therefore, the specimen with wrinkle defects under a reverse impact fails earlier during the compression process, resulting in significantly lower residual compressive strength after impact.

In summary, except for the slight increase in the residual compressive strength of the specimen with the wrinkle at the third layer, which is attributed to the stronger suppression of impact damage by the wrinkle fibers, specimens with wrinkle defects all show a certain degree of reduction in their residual compressive strength after impact, compared to that of the specimen without wrinkle defects. Moreover, the amplitude of the reduction in the residual compressive strength mainly depends on the matrix damage and delamination after impact. The significant matrix damage and delamination after impact result in a larger reduction in residual compressive strength.

### 4.2. Damage Analysis

**Figure 12:**
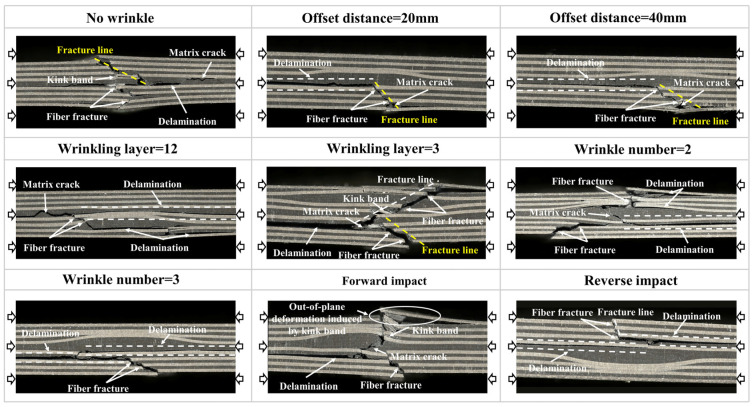
Damage analysis and CAI test of specimens with different defects.

The typical failure morphologies of various specimens with wrinkle defects after the compression test are shown in Figure 12. Obviously, the specimen without wrinkle defects exhibits a typical compression failure mode. During the compression process, delamination occurs at the center of the specimen caused by the impact and expands laterally under the compressive load, and the fibers form kink bands under the interlayer shear forces. Due to the relatively weak compressive properties of the fibers, the kink band facilitates crack propagation, ultimately resulting in fiber fractures. Furthermore, the distinct fracture angle observed in the specimen indicates that the specimen bears a large compressive load and fails through the thickness direction. When the specimen contains offset wrinkles, the failure modes of the specimens with offset distances of 20 mm and 40 mm are quite similar. After impact, the delamination at the center of the specimen extends laterally during the compression process. This results in fiber fracture and matrix failure occurring at the wrinkle fibers, leading to the overall compressive failure of the specimen. Moreover, due to the large area of delamination, the specimen has not yet exhibited failure through the thickness direction. As a result, the residual compressive strength of the specimen is significantly reduced compared to that of the specimen without wrinkle defects, as described in Section 4.1. Similarly, as for the specimen with the wrinkle at the 12th layer, a large area of delamination occurs at the center of the specimen during the compression process, and the fiber fracture occurring at the wrinkle fibers causes the outward propagation of the matrix crack, leading to the premature compressive failure of the specimen. Consequently, the compressive strength of the specimen also shows a significant decrease compared to that of the specimen without wrinkle defects. In contrast, when the wrinkle is located at the third layer, due to the larger extent to which the wrinkle fibers are subjected to the impact load, the matrix damage and delamination after impact are relatively small, as described in Section 3.3. During the compression process, the specimen retains a high load-bearing capacity, resulting in a distinct fracture angle and failure through the thickness direction, which contributes to higher residual compressive strength. When the specimen contains two wrinkles, delamination between the two wrinkles and fiber fracture at the wrinkles under the compression process lead to crack propagation along the thickness direction of the specimen. When the specimen contains three wrinkles, a larger area of delamination occurs between the wrinkles under the compression process, leading to the premature failure of the specimen. Consequently, the specimen exhibits lower residual compressive strength. As for specimens under a forward impact, due to the wrinkle fibers being subjected to the impact load, the matrix damage and delamination after impact are relatively small. Therefore, the specimen still undergoes failure through the thickness direction under a compressive load, resulting in only a small reduction in the residual compressive strength. On the other hand, as for specimens under a reverse impact, due to the wrinkle fibers not being subjected to the impact load, there is relatively large matrix damage and delamination after impact. This results in a larger area of delamination occurring at the center of the specimen under a compressive load, leading to the premature failure of the specimen. The residual compressive strength of the specimen under a reverse impact decreases significantly.

To summarize, compared to the specimen without wrinkle defects, the significant reduction in the residual compressive strength of specimens with various types of wrinkle defects is mainly due to the premature failure of the specimens caused by the large area of delamination under a compressive load.

## 5. Conclusions

In this study, the effects of wrinkle defects on the impact response and residual compressive strength after impact in CFRP specimens is evaluated. The impact response and compressive properties after impact in CFRP specimens with different wrinkle defects are investigated through experiments and numerical simulations. Four key variables related to wrinkle defects are examined: the wrinkle offset distance, wrinkling layer, wrinkle number, and impact direction.

It can be concluded that the wrinkle defect mainly affects the impact and compressive response via the wrinkle fibers being subjected to impact stress induced by the impact load and the wrinkle stress concentration. When a single wrinkle is located at the impact point and close to the impact surface under a forward impact direction, the wrinkle defect shows a minimal effect on the impact response and compressive performance. Otherwise, the wrinkle defect has a greater effect on the impact response and compressive performance, with a maximum reduction of residual compressive strength of up to approximately 27.50% for specimens under the reverse impact direction.

In summary, to avoid the effects of wrinkle defects on the impact properties of CFRP structures, if the appearance of wrinkle defects cannot be avoided in the manufacturing process, it should be ensured that the position of the wrinkle defects is close to the impact surface of the structure and the convex side of the wrinkle is towards the impact surface under the premise of reducing the number of wrinkles as much as possible.

## Figures and Tables

**Table 1:** Test arrangement.

ID	Wrinkle Offset Distance (mm)	Wrinkling Layer	Wrinkle Number	Impact Direction
L-NW	-	-	-	Forward
L-LW-O1	20	8	1	Forward
L-LW-O2	40	8	1	Forward
L-LW-L1	0	12	1	Forward
L-LW-L2	0	3	1	Forward
L-LW-N2	0	8	2	Forward
L-LW-N3	0	8	3	Forward
L-LW-F	0	8	1	Forward
L-LW-R	0	8	1	Reverse

**Table 2:** Material properties of T700/7901 [31].

*E*_1_ /GPa	*E*_2_ /GPa	*E*_3_ /GPa	*ν*_12_	*ν*_13_	*ν*_23_	*G*_12_/GPa	*G*_13_ /GPa	*G*_23_ /GPa	*X*_T_ /MPa	*X_C_* /MPa
120	8	8	0.25	0.25	0.33	4.5	4.5	3	1600	1200
*Y*_T_ /MPa	*Y*_C_ /MPa	*Z*_T_ /MPa	*Z*_C_ /MPa	*S*_12_ /MPa	*S*_13_ /MPa	*S*_23_ /MPa	*G*_1t_ N/mm	*G*_1c_ N/mm	*G*_2t_ N/mm	*G*_2c_ N/mm
55	200	55	200	100	100	90	133	40	0.3	1

**Table 3:** Parameters of cohesive elements.

Parameter	Value
Stiffness (MPa)	*K*_nn_ = *K*_ss_ = *K*_tt_ = 50,000
Strength (MPa)	*σ*_nn_ = 35, *σ*_ss_ = *σ*_tt_ = 60
Fracture toughness (N/mm)	*G*_nn_ = 0.6, *G*_ss_ = *G*_tt_ = 2.1

**Table 4:** Residual compressive strength of CFRP with different wrinkle defects.

ID	Offset Distance (mm)	Wrinkling Layer	Wrinkle Number	Impact Direction	Strength (MPa)	Variation
L-NW	-	-	-	Forward	174.79 ± 8.15	-
L-LW-O1	20	8	1	Forward	148.45 ± 15.23	−15.10%
L-LW-O2	40	8	1	Forward	161.06 ± 13.62	−7.90%
L-LW-L1	0	12	1	Forward	129.32 ± 10.08	−26.00%
L-LW-L2	0	3	1	Forward	183.24 ± 12.36	4.80%
L-LW-N2	0	8	2	Forward	167.38 ± 9.87	−4.20%
L-LW-N3	0	8	3	Forward	128.65 ± 12.81	−26.40%
L-LW-F	0	8	1	Forward	169.81 ± 19.14	−2.90%
L-LW-R	0	8	1	Reverse	126.65 ± 17.49	−27.50%

**Figure A1:**
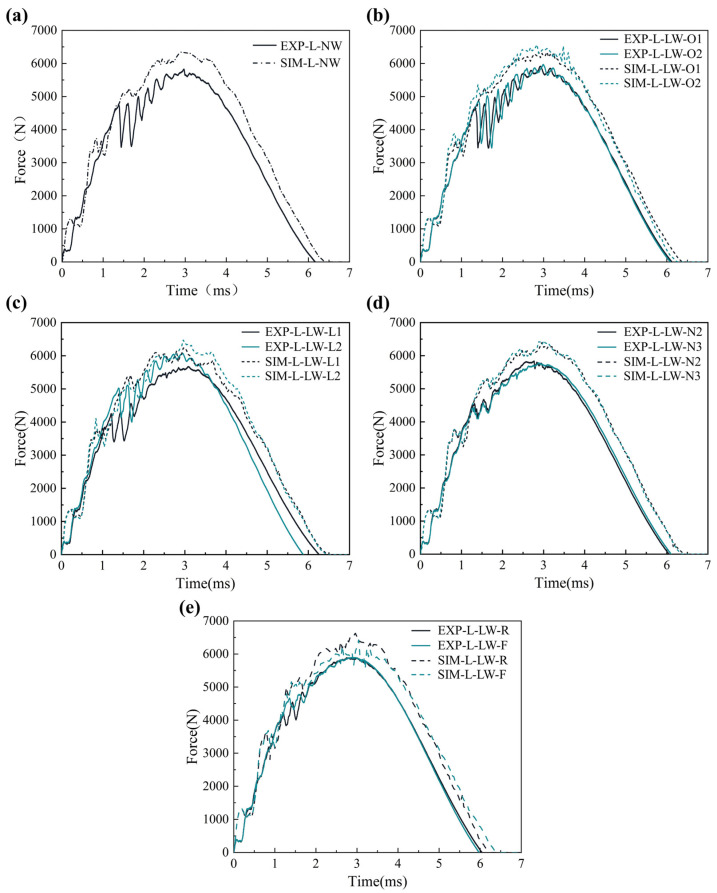
Experimental and simulation results regarding impact response of specimens (**a**) without wrinkles and with different wrinkle defects: (**b**) offset distance; (**c**) wrinkling layer; (**d**) wrinkle number; (**e**) impact direction.

## Data Availability

Data available on request due to restrictions (e.g., privacy, legal, or ethical reasons).

## Symbols and Abbreviations

*C*	curvature percentage of specimen
CAI	compression after impact
CFRP	carbon fiber-reinforced polymer
*E*_1_, *E*_2_, *E*_3_	elastic modulus of composite
*F*	compression load
*G*_12_, *G*_13_, *G*_23_	shear modulus of composite
*G*_1*t*_, *G*_1*c*_	0° fracture toughness of composite
*G*_2*t*_, *G*_2*c*_	90° fracture toughness of composite
*G_nn_, G_ss_, G_tt_*	fracture toughness of cohesive model
*h*	wrinkle amplitude
*h_s_*	thickness of compressive specimen
*k*	wrinkle ratio
*K_nn_, K_ss_, K_tt_*	stiffness of cohesive model
*L*	wrinkle width
*S*_12_, *S*_13_, *S*_23_	shear strength of composite
*w*	width of compressive specimen
*X_T_, X_C_*	0° strength of composite
*Y_T_, Y_C_*	90° strength of composite
*Z_T_, Z_C_*	out-of-plane strength of composite
*ε_1_, ε_2_*	surface strain of specimen
*ν*_12_, *ν*_13_, *ν*_23_	Poisson’s ratio of composite
*σ_CAI_*	residual compressive strength
*σ_nn_, σ_ss_, σ_tt_*	strength of cohesive model

## Appendix A

In this study, to verify the accuracy of the numerical simulation model, the impact simulation results of all specimens are compared with the experimental results. The comparison results are shown in Table A1 and Figure A1. The results indicate that the errors between the experimental and the simulation results of all specimens are less than 15%, indicating that the numerical model has good reliability and accuracy.

**Table A1:** Comparison between experimental and simulation results regarding impact response.

Specimen ID	Maximum Contact Force (N)		
	Experiment	Simulation	Error
L-NW	5895.43	6334.56	7.45%
L-LW-O1	5923.10	6296.07	6.30%
L-LW-O2	5946.52	6468.11	8.77%
L-LW-L1	5679.55	6236.76	9.81%
L-LW-L2	6109.57	6421.03	5.10%
L-LW-N2	5879.21	6396.62	8.80%
L-LW-N3	5797.89	6442.37	11.12%
L-LW-F	5934.73	6368.15	7.30%
L-LW-R	5899.54	6272.70	6.33%
